# All-suture anchors for distal biceps tendon repair: a preliminary outcome study

**DOI:** 10.1007/s00402-022-04690-0

**Published:** 2022-11-22

**Authors:** Sebastian Lappen, Stephanie Geyer, Pavel Kadantsev, Maximilian Hinz, Benjamin Kleim, Hannes Degenhardt, Andreas B. Imhoff, Sebastian Siebenlist

**Affiliations:** Department of Orthopedic Sports Medicine, Technical University of Munich, Ismaninger Str. 22, 81675 Munich, Germany

**Keywords:** Biceps, Distal biceps rupture, Anatomic biceps repair, Suture anchor, Tendon injuries

## Abstract

**Introduction:**

The aim of this study was to retrospectively evaluate the clinical outcome of double intramedullary all-suture anchors’ fixation for distal biceps tendon ruptures.

**Materials and methods:**

A retrospective case series of patients who underwent primary distal biceps tendon repair with all-suture anchors was conducted. Functional outcome was assessed at a minimum follow-up of at 12 months based on the assessments of the Mayo Elbow Performance Score (MEPS), Andrews–Carson Score (ACS), Quick Disabilities of the Arm, Shoulder, and Hand questionnaire (QuickDASH), and the Visual Analog Scale (VAS) for pain. Maximum isometric strength test for flexion and supination as well as postoperative range of motion (ROM) were determined for both arms.

**Results:**

23 patients treated with all-suture anchors were assessed at follow-up survey (mean age 56.5 ± 11.4 years, 96% male). The follow-up time was 20 months (range *Q*_0.25_–*Q*_0.75_, 15–23 months). The following outcome results were obtained: MEPS 100 (range *Q*_0.25_–*Q*_0.75_, 100–100); ACS 200 (range *Q*_0.25_–*Q*_0.75_, 195–200); QuickDASH 31 (range *Q*_0.25_–*Q*_0.75_, 30–31); VAS 0 (range *Q*_0.25_–*Q*_0.75_, 0–0). The mean strength compared to the uninjured side was 95.6% (range *Q*_0.25_–*Q*_0.75_, 80.9–104%) for flexion and 91.8 ± 11.6% for supination. There was no significant difference in ROM or strength compared to the uninjured side and no complications were observed in any patient.

**Conclusion:**

Distal biceps tendon refixation using all-suture anchors provides good-to-excellent results in terms of patient-reported and functional outcome. This repair technique appears to be a viable surgical option, although further long-term results are needed.

**Level of evidence:**

Level IV (case series)

## Introduction

Ruptures of the distal biceps tendon are the most common tendon ruptures of the elbow joint with an incidence of 1.2–2.55 per 100,000 patient-years [1]. Male, physically active patients between the fourth and sixth decade of life are most often affected [1, 2]. Due to possible impairment in elbow flexion and supination strength and strength endurance, conservative therapy is rarely indicated and surgery is generally considered the treatment of choice [3]. A variety of anchor systems are currently available for biceps tendon repair [4–7]. Though, there remains controversy about the optimal fixation technique. The most common fixation systems are suture anchors, cortical buttons, and interference screws [8]. Until now though, no significant advantage of either method could be shown over another in clinical studies [9–11].

Recently, all-suture anchors were introduced as an alternative method for distal biceps tendon refixation [12, 13] as they show some theoretical advantages. First, their low-profile construct and small drill holes are potentially less traumatic to the cortex [14, 15], and second, no metallic artifacts are generated in magnetic resonance imaging (MRI) distorting the postoperative assessment. Biomechanically, all-suture anchors show equivalent properties with a similar pull-out strength and load to failure as solid titanium anchors [16]. To date, however, no study investigating the clinical outcome following refixation of the distal biceps tendon using all-suture anchor has been published.

Therefore, the aim of this study was to evaluate the preliminary clinical outcome of the double intramedullary fixation using all-suture anchors in patients with acute distal biceps tendon ruptures. It was hypothesized that (1) functionality would be restored consistent with the uninjured side and that (2) a less overall complication rate will be achieved when compared to established techniques.

## Materials and methods

### Patient selection

A retrospective chart review was performed on patients who received intramedullary fixation of the distal biceps tendon using all-suture anchors between February 2017 and May 2020 at the author’s institution. Patients were included with traumatic primary distal biceps tendon rupture, age over 18 years, and a minimum follow-up of 12 months. Patients with chronic distal biceps tendon ruptures (longer than 30 days between injury and surgery), previous surgeries of the affected elbow or relevant comorbidities such as diseases of the rheumatic type, cervical and peripheral neuropathies, malignant tumor diseases, and metabolic diseases were excluded. Additional information such as demographic data including age, sex, and hand dominance as well as previously practiced sports occupation and risk factors such as smoking or steroid abuse collected. Surgical charts were analyzed for surgical technique and intraoperative and postoperative complications, as well as rehabilitation protocol. Institutional review board approval was obtained prior to this study (268/20-S) and the study was conducted according to the Declaration of Helsinki. Informed consent was obtained by each individual prior to the clinical evaluation.

### Surgical management

**Fig. 1:**
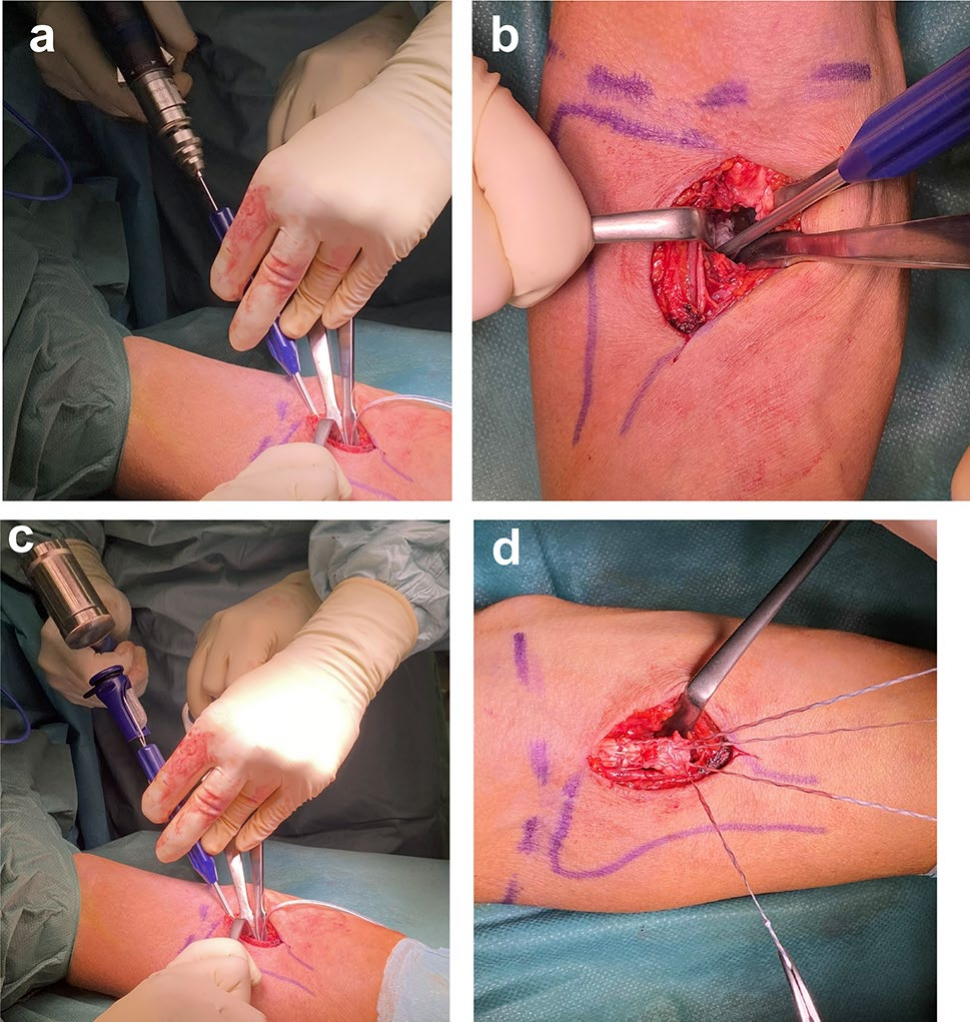
Insertion of the all-suture anchor. **a** Drilling into the anterior cortex at an entry angle of 45° for placement of the all-suture anchor. **b** An all-suture anchor is now inserted into the radial tuberosity. **c** A second all-suture anchor is inserted distally to the first one. **d** With the anchors inserted into the bone, the distal biceps tendon can now be augmented

All surgeries were performed by one fellowship trained elbow surgeon (S.S.). The radial tuberosity was approached according to the technique described by Siebenlist et al. [4]. In all cases, refixation was performed with two all-suture anchors (FiberTak DX; Arthrex Inc., Naples, FL, USA) that were inserted at the intramedullary cavity of the proximal and distal radial tuberosity as it was similarly described for button fixation [17]. To implant the anchors, two 1.6 mm unicortical holes were drilled into the anterior cortex without perforating the posterior cortex. The drillings were set at a distance of 10 mm and an entry angle of 45° (Fig. 1). With one end of each SutureTape of the anchor, the tendon stump was augmented using interlocking Krackow stitches. The other suture limb was simply pierced back through the tendon. With the elbow in 30° flexion and full supination, the tendon was fixed onlay to the radial tuberosity by pulling both free suture limbs. The suture ends were then tied down to the tuberosity. Postoperative radiographs were taken in all patients on the first day after surgery.

All patients were treated with a standardized postoperative protocol. They were postoperatively immobilized for 2 days in a cast and a hinged brace was subsequently applied for 6 weeks. The brace was limited to 20° extension to protect the tendon repair from maximum extension loads for 2 weeks postoperatively. Physical therapy with free active motion was allowed from the first postoperative day. Six weeks after the operation, the brace was removed, and active biceps muscle strengthening was started.

### Outcome evaluation

Functional outcome was assessed using the Mayo Elbow Performance Score (MEPS), the Andrews–Carson Score (ACS), and the shortened questionnaire on disabilities of the arm, shoulder and hand (QuickDASH). The current pain status was assessed using a visual analog scale (VAS) with a rating range from 0 (“no pain”) to 10 (“worst imaginable pain”).

Sporting activities of the patients before the rupture and at follow-up were queried to determine the practice of high-demanding sports, such as weightlifting and the return-to-sport rate.

Clinical assessment consisted of the standardized measurement of the range of motion (ROM) of both elbows using a goniometer. Isometric muscle strength tests (IsoBex; MDS Medical Device Solutions AG, Oberburg, Switzerland) were performed on both arms. The maximum strength of flexion was measured at 90° elbow flexion and forearm supination, the maximum supination strength at 90° elbow flexion with a neutral forearm. Three consecutive measurements were obtained for each patient and then averaged. To accurately assess postoperative differences in flexion and supination strength relative to the non-operated side, strength ratios were calculated for each patient. For this purpose, flexion and supination strength of the operated side was divided by the respective strength of the non-operated side (limb-symmetry index), with final values being presented as percentage. Outcome measures and strength testing were performed by a single examiner not involved in the surgical management (S.L.).

### Statistical analysis

The sample size was calculated using the G*Power software (latest version 3.1.9.7; Heinrich Heine Universität Düsseldorf, Düsseldorf, Germany). Using the effect size in terms of supination strength compared to the uninjured side, referring to data previously published by Siebenlist et al. [18], Wilcoxon–Mann–Whitney test was set to calculate sample size. α was set at 0.05. The total samples size of 20 was required to achieve power of 0.9.

All calculations were performed with SPSS Statistics (Version 25, Property IBM Corp., NY, USA). Descriptive statistics were used for continuous variables. Normal distribution was assessed by the Shapiro–Wilk test. Normally distributed values were described by mean and standard deviation, skewed distributed values by median and interquartile range (range *Q*_0.25_–*Q*_0.75)._ Paired *t* test (for normally distributed data) and Wilcoxon tests (for non-normally distributed data) were used to assess differences between the affected and unaffected sides in range of motion and strength based on a limb-symmetry index (affected/unaffected). A value of *p* < 0.05 was considered significant.

## Results

### Demographics

Of 28 patients who met the inclusion criteria, 23 (82%) could be included in the current study. Five patients were lost to follow-up. Four of the included patients were unable to return for clinical follow-up and were therefore evaluated by telephone interview. The median follow-up was 20 months (range *Q*_0.25_–*Q*_0.75_, 15–23 months). Patient population consisted of 22 male and one female patients with an average age of 56.5 ± 11.4 years. The mean interval between rupture and surgical treatment was 16.5 ± 4.8 days. Nine patients reported participating in high-demanding sports such as weightlifting, material arts, or throwing sports with none of the patients reporting a history of anabolic steroids’ intake.

### Outcome evaluation

The results of the scoring systems are summarized in Table 1. All but two patients achieved good-to-excellent results regarding functional outcome scores and were able to return to their preoperative level of sports (96%). One patient scoring a DASH of 66 reported a painful concomitant shoulder pathology at follow-up which also prevented him from returning to his preoperative level of sports. According to the patient, however, none of his symptoms were caused by his elbow, and he achieved excellent results in MEPS (100 points) and ACS (200 points) with no elbow pain whatsoever (VAS of 0 points). A second patient merely achieved satisfactory results in DASH (57 points), MEPS (70 points), and ACS (170 points). The patient stated that he still experienced exercise-related elbow pain with a VAS up to 6 points but was nevertheless satisfied with the overall result of the surgery. He achieved full ROM and equivalent strength values compared to the uninjured side and was able to fully return to his preoperative level of exercise.

**Table 1:** Functional outcome

Parameter	Value*
MEPS	100 (100–100)
DASH	31 (30–31)
Sports/performing arts	4 (4–4)
Work	4 (4–4.5)
ACS	200 (195–200)
VAS	0 (0–0)

*MEPS* Mayo elbow performance score, *DASH* disabilities of the arm, shoulder and hand, *ACS* Andrew–Carson score, *VAS* visual analog scale

*The values are given as the median (25th percentile–75th percentile)

**Fig. 2:**
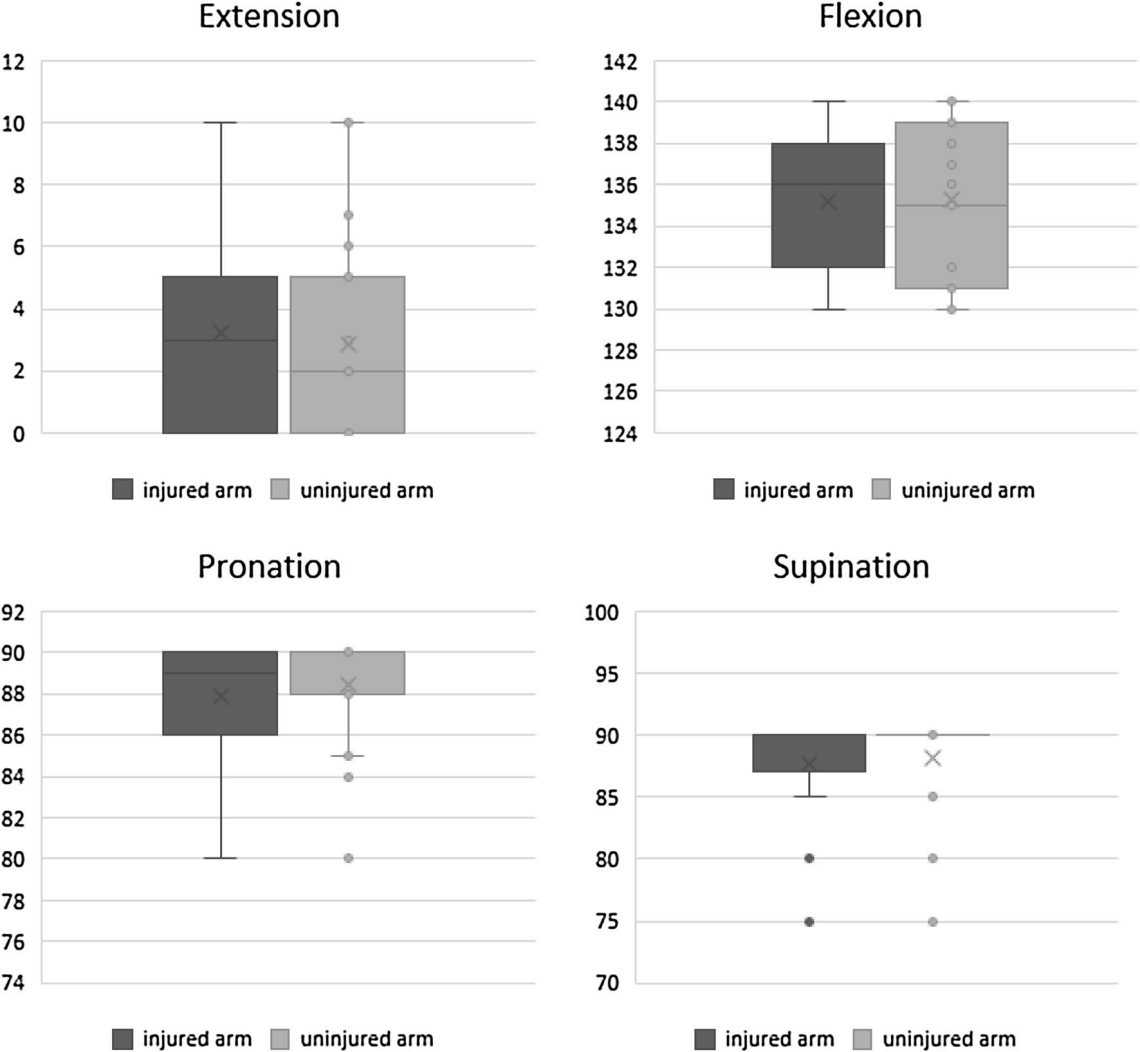
Range of motion of the injured arm compared to the uninjured arm applied in degrees (°)

The mean postoperative flexion was 136° (range *Q*_0.25_–*Q*_0.75,_ 132–138°), for extension 3° (range *Q*_0.25_–*Q*_0.75,_ 0°-5°), for supination 90° (range *Q*_0.25_–*Q*_0.75,_ 87°–90°), and for pronation 89° (range *Q*_0.25_–*Q*_0.75_, 86°–90°). There was no significant difference of ROM to the uninjured side (n.s.). Figure 2 gives an overview over the ROM of the injured arm compared to the uninjured arm.

**Fig. 3:**
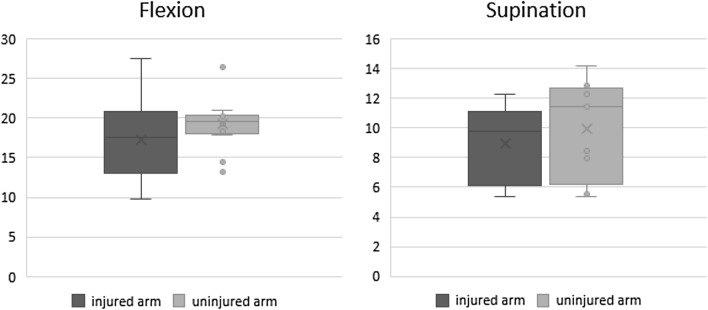
Maximum isometric strength for both the injured and the uninjured arm applied in Newton (*N*)

The mean strength of the operated arm compared to the uninjured side was 95.6% (range *Q*_0.25_–*Q*_0.75_, 80.9–104%) for flexion and 91.8% ± 11.6% for supination. The differences in strength between the dominant and non-dominant arms were negligible (n.s.). Figure 3 gives an overview over the strength of the injured arm compared to the uninjured arm.

**Fig. 4:**
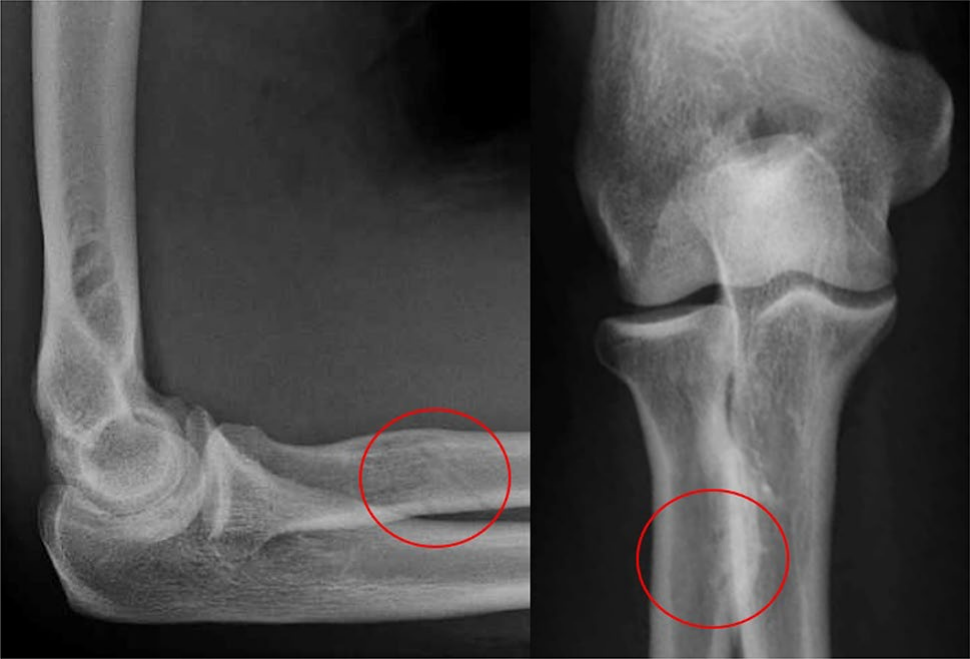
The drill holes in the radial tuberosity can be seen on the postoperative X-rays

No intra- or postoperative complications such as infections or nerve injuries were observed. None of the postoperative X-ray controls performed on all patients showed evidence of bony injuries (Fig. 4). No surgical revisions were needed. Pain issues or functional restrictions related to HO were not seen in any case.

## Discussion

The most important finding of the present study is that distal biceps tendon repair using all-suture anchors represents a novel method option showing reliable results regarding elbow flexion and supination strength as well as good-to-excellent functional outcome scoring.

The preferable surgical technique for distal biceps tendon repair remains controversial and the risk of complications is still an important factor. The present study showed no complications in evaluated patients who had received refixation of the distal biceps tendon with all-suture anchors in a single-incision technique. Single-incision techniques show an overall lower complication rate compared to double-incision techniques [19]. One advantage of single-incision techniques is the overall lower risk of heterotopic ossifications (HO) [20, 21]. The formation of HO is promoted by mesenchymal progenitor cell (MPC) recruitment, which can emerge from an open medullary canal [22]. The risk for HO might therefore be reduced using all-suture anchors as the drill hole is blocked by the suture bundle inserted into the bone and MPC recruitment can be prevented. Furthermore, postoperative stiffness is seen more frequently in double-incisions approaches [19] and supination strength can be affected, as damage to the supinator muscle from the posterior incision can reduce the muscle’s strength [23]. The present study showed excellent results in ROM and supination and flexion strength, similarly to other single-incision techniques [17, 24, 25]. A general disadvantage of single-incision techniques is the higher rate of lateral antebrachial cutaneous nerve (LACN) neuropraxia compared to double-incision techniques [19]. However, according to a systematic review by Kodde et al., this only occurs in 0.3% of cases and is mostly transient [26]. No LACN lesions were seen in the present study. This might be explained by the less invasiveness of the described technique as less soft-tissue compression through the use of surgical hooks is required due to the cannulated drilling capability. However, a larger patient population is needed to support this possible explanation.

A variety of anchor systems, such as cortical buttons, suture anchors, and interference screws, are currently available for distal biceps tendon repair [4–7]. There is currently no consensus whether tendon-to-bone healing is better facilitated by tendon fixation in a bone tunnel or on a cortical surface. Tendon fixation techniques in a bone tunnel, such as cortical buttons and interference screws, are widely utilized as they show strong initial fixation strength [27, 28]. In addition, intramedullar button fixation devices such as the ones described by Siebenlist et al. [4, 17] or Caekebeke et al. [29] allow reinsertion of the biceps tendon using a single-incision approach. However, an investigation in a rabbit model by Tan et al. showed similar tendon healing profiles within a bone tunnel compared with direct tendon–bone healing of biceps tenodesis [30]. Among onlay fixation techniques, suture anchors are widely utilized as they show various advantages: they are considered to be easy to use and the risk of iatrogenic injury to the posterior interosseous nerve is minimized, since they do not require bicortical drilling [12]. However, revision surgery can be difficult to perform, since solid anchors often need to be removed by overdrilling with significant bone loss at the tuberosity. And moreover, follow-up examinations by MRI are not practicable due to limited informative value when metal anchors have been used. Thus, distal biceps tendon repair with all-suture anchors presents a reasonable alternative to minimize these disadvantages. Since the drill holes for the placement of all-suture anchors require a smaller diameter compared to other fixation techniques, bone loss is reduced and greater intraoperative flexibility is made possible, especially for revision cases [13]. Furthermore, biomechanical studies show all-suture anchors to have similar ultimate failure loads and stiffness to unicortical intramedullary buttons [31] and titanium anchors [16]. These findings may be supported by the present clinical results, since no recurrent rupture was observed at the final follow-up examination.

Although time-zero mechanical strength of distal biceps tendon repair using all-suture anchors appears to be comparable to other anchor systems, clinical data are still limited. The postoperative mean range of motion determined in the present study was similar to those determined in a meta-analysis by Litwoski et al. [32]. Similar improvement was found in the isokinetic force measurements. With a flexion strength of 95.6% and a supination strength of 91.8% of the uninjured side, these values were above the mean values of 82.7–84 and 85.2–89.3% determined by Litwoski et al. [32]. However, clinical results concerning distal biceps tendon repair using all-suture anchors are still rare. While Woodall et al. [13] and Cross et al. [12] described the surgical technique of distal biceps tendon repair with all-suture anchors as case reports, no clinical outcome was described by these authors until now. However, all-suture anchors are already being used successfully in other locations such as the shoulder and the hip [33–35].

However, fixation with all-suture anchors is not without limitations. Biomechanical studies examining all-suture anchors showed them to be susceptible to micromotion and early gap formation [15]. In addition, an in vivo animal study found that labrum repairs using all-suture anchors can result in cyst-like cavities with a border of dense lamellar bone at the anchor points, indicating that all-suture anchors are at risk of clinical failure [36]. However, this has not been observed in human studies and has still not been investigated for the repair of the distal biceps tendon [37, 38]. Since the patients included in the present study were not examined radiologically at follow-up, such cyst-like cavities cannot be completely ruled out. However, none of the patients experienced complaints that suggested such cavities, just as there was no rerupture.

The present study has some limitations, such as its retrospective design, the follow-up rate of 82% of treated patients (including 4 patients only evaluated by telephone interview), the short-term follow-up, and the relatively small sample size. However, other clinical outcome studies included a comparable number of patients [17, 24, 25, 39].

The strengths of the study include that subjective and objective outcome measures, including functional evaluation and isometric strength tests, are presented. Even though, this novel fixation technique has been described in technical notes [12, 13], to the best of our knowledge, this is the first cohort study reporting about clinical outcome of all-suture anchor repair for distal biceps tendon ruptures. We therefore believe that the promising clinical outcome confirms our hypothesis that all-suture anchors can be used as an effective and safe method to restore distal biceps tendon ruptures. However, larger clinical studies are needed to confirm the clinical findings and identify possible complications.

## Conclusion

Distal biceps tendon repair by intramedullary refixation using all-suture anchors provides good-to-excellent results in terms of clinical outcome, ROM, and restoration of strength. The data shown in the present study confirm this novel technique as an effective alternative to established procedures for repairing the distal biceps tendon with a very low complication rate.
